# Supplementary material for: The effects of genetic and modifiable risk factors on brain regions vulnerable to ageing and disease
Source: Nat Commun. 2024 Mar 27;15:2576. doi: 10.1038/s41467-024-46344-2 (PMC10973379; doi:10.1038/s41467-024-46344-2)
Supplement: Supplementary file 1 — Supplementary Information [file 41467_2024_46344_MOESM1_ESM.pdf]

Supplementary Information

BrainMap behavioural analysis

In order to better characterise the LIFO brain network, a *post hoc* 'behavioural' analysis was performed as implemented in the dedicated plug-in for the software Mango[1]. This tool is based on the functional section of the BrainMap database[2] and provides a quantitative association between user-defined regions-of-interest (ROI) - in our case, the LIFO brain network - and 60 functional maps of behavioural sub-domains, organised in 5 classes: perception, interoception, emotion, cognition and action. Briefly, the behavioural association is computed observing the spatial intersection between the ROI and the probability density function of each sub-domain. Hypothesis testing is then computed against the probability of activation randomly falling inside the ROI. Only sub-domains with a Z-score ≥ 3 are deemed to be significant[1]. Behavioural analysis is particularly sensitive to the thresholding applied to the input map, as every non-zero voxel is considered the same (whether a minimally significant one or a local maximum). We thus compared the results obtained for the LIFO brain network thresholded both at Z = 4 and Z = 10.

UK Biobank modifiable risk factors (MRFs)

When both 'instance 0' (i.e. variables collected at the recruitment of the subject) and 'instance 2' (i.e. variables collected at the MRI acquisition visit) were available they were both included and treated as separate variables.

'Do not know' and 'Prefer not to answer' responses, whenever present, were treated as missing.

Nested variables were resolved based on UK Biobank information. For example, subjects who answered "Never" for the variable "Frequency of drinking alcohol" were originally coded as having missing values for the subsequent variable "Frequency of consuming six or more units of alcohol". To resolve this issue, subjects who never drink alcohol were also coded as subjects never drinking six or more units of alcohol. Similarly, subjects who answered "No" for the variables "Ever had prolonged feelings of sadness or depression" and "Ever had prolonged loss of interest in normal activities" were originally coded as having missing values in the subsequent variable "Lifetime number of depressed periods". For the purpose of our study, these values were instead recoded as "0" for the last question.

Categorical variables were transformed into binary, either by merging the same variables together (e.g. regularly takes medication for diabetes, a question that is asked separately for each gender), collapsing similar set of answers within the same question (e.g. leisure/social activities: attending gym OR pub OR education class, etc.) or splitting the original variable with a given number of x answers possible into the same x number of binary variables (e.g. leisure/social activities: gym ONLY, pub ONLY, education class ONLY, etc.).

Probability for two hits to be in PAR1

If our 7 significant hits can be found anywhere across the whole genome, the probability that 2 out of 7 are in PAR1 is as follows:

g = length of human genome in bp: 3,053,521,184

h = length of PAR1 in bp: 2,639,519

(g and h in h19 lengths accounting for double counting or not counting some of Y)

Chance (as a probability between 0 and 1): (h/g)^2×(1-h/g)^5 = $\mathbf{7.4\times10^{-7}}$

In addition, if we consider as null hypothesis that "hits are distributed between PAR1 and the rest of the genome according to the probabilities implied by a uniform distribution over all loci on the genome, or that hits are more likely to arise in the rest of the genome rather than in PAR1" (i.e., under H_0 each hit has a probability <= h/g of being in PAR1, and under the alternate hypothesis each hit has a probability > h/g of being in PAR1), the frequentist test for this situation is the binomial test (one tailed, with alternative greater), with 7 trials, 2 successes, and probability of success h/g. It returns, when implemented in R: **$P = 1.56 \times 10^{-5}$**

Testing for differences in the reduced sample of n = 35,527

In order to verify possible sub-sampling bias induced by the selection of the complete cases, two-sample Kolmogorov-Smirnov test was used to compare the distributions. None of the modifiable risk factors (n = 12) from the Stage 2 analysis were significantly different between the original sample and the reduced, complete sample after correction for multiple comparisons across those factors: diabetes diagnosed by doctor $P_{uncorr} = 1$, nitrogen dioxide air pollution in 2005 $P_{uncorr} = 0.713$, alcohol intake frequency $P_{uncorr} = 0.713$, sleep duration $P_{uncorr} = 0.997$, waist circumference $P_{uncorr} = 0.021$, past tobacco smoking $P_{uncorr} = 1$, medication for blood pressure $P_{uncorr} = 1$, frequency of stairs climbing in last 4 weeks $P_{uncorr} = 0.882$, hearing difficulty/background $P_{uncorr} = 1$, medication for pain relief $P_{uncorr} = 1$, pub or social clubs $P_{uncorr} = 1$, medication for cholesterol $P_{uncorr} = 1$.

Modifiable risk factors two-stage analysis

There is substantial redundancy within each MRF category. Moreover, not all UK Biobank participants provided data for all variables; an analysis limited to those with complete data would be biased, and based on a small, low-powered sample.

We addressed both issues via a two-stage analysis in which first we identified which variable within a category best represents eventual associations of that category with the LIFO brain network loadings. Once this had been established, we investigated the unique contribution of that category, over and above all other categories, to the LIFO loadings, while comprehensively correcting for multiple testing with the conservative Bonferroni method.

While the second stage consisted of only one model, that was one of a large set of models that *could* have been investigated if complete data were available and any single (i.e., not necessarily the "best") variable were allowed to be used to represent each of the 15 MRF categories in the second stage. The number of such tests is thus:

$$N = \prod_{k=1}^{15} N_k$$

where: N_k = total number of MRFs per category, k = category.

Selection of the "best" variable provided an algorithmic shortcut that bypassed the need for these many tests and further addressed issues related to missingness. However, correction for these many tests was necessary, otherwise the screening provided by the first stage would render the analysis circular. While these many tests are not independent, we again took the conservative Bonferroni method, thus with a two-tailed significance cut-off of $P = 0.05/(2\times N) = 0.05/[2\times(5.41\times10^{14})] = 4.62\times10^{-17}$.

References

1. Lancaster, J. L. et al. Automated regional behavioral analysis for human brain images. Front Neuroinform 6, 23, doi:10.3389/fninf.2012.00023 (2012).

2. Fox, P. T. & Lancaster, J. L. Opinion: Mapping context and content: the BrainMap model. Nature reviews. Neuroscience 3, 319-321, doi:10.1038/nrn789 (2002).

Full output of the mediation analyses on the dominant and recessive models

Lead bi-allelic variant from cluster 5 on Alzheimer's disease via LIFO brain network

Causal Mediation Analysis

Dominant analysis:

Nonparametric Bootstrap Confidence Intervals with the Percentile Method
(Inference Conditional on the Covariate Values Specified in `covariates')

	Estimate 95%	CI Lower 95%	CI Upper	p-value
ACME (control)	1.00e-04	4.13e-05	1.81e-04	4e-05 ***
ACME (treated)	1.33e-04	5.22e-05	2.44e-04	4e-05 ***
ADE (control)	4.33e-04	-5.85e-04	1.52e-03	0.41
ADE (treated)	4.66e-04	-6.31e-04	1.63e-03	0.41
Total Effect	5.66e-04	-5.02e-04	1.71e-03	0.31
Prop. Mediated (control)	1.77e-01	-1.77e+00	2.07e+00	0.31
Prop. Mediated (treated)	2.35e-01	-1.56e+00	1.99e+00	0.31
ACME (average)	1.16e-04	5.19e-05	1.99e-04	4e-05 ***
ADE (average)	4.50e-04	-6.08e-04	1.57e-03	0.41
Prop. Mediated (average)	2.06e-01	-1.66e+00	2.03e+00	0.31

Signif. codes: 0 '***' 0.001 '**' 0.01 '*' 0.05 '.' 0.1 ' ' 1
Sample Size Used: 22128
Simulations: 50000

Recessive analysis:

Nonparametric Bootstrap Confidence Intervals with the Percentile Method
(Inference Conditional on the Covariate Values Specified in `covariates')

	Estimate 95%	CI Lower 95%	CI Upper	p-value
ACME (control)	1.46e-04	5.35e-05	2.80e-04	4e-05 ***
ACME (treated)	1.64e-04	3.49e-11	5.26e-04	4e-05 ***
ADE (control)	1.92e-04	-1.76e-03	2.95e-03	0.94
ADE (treated)	2.11e-04	-1.93e-03	3.26e-03	0.94
Total Effect	3.56e-04	-1.76e-03	3.40e-03	0.86
Prop. Mediated (control)	4.09e-01	-1.01e+00	1.44e+00	0.86
Prop. Mediated (treated)	4.61e-01	-8.21e-01	1.40e+00	0.86
ACME (average)	1.55e-04	3.96e-05	3.74e-04	4e-05 ***
ADE (average)	2.01e-04	-1.84e-03	3.11e-03	0.94
Prop. Mediated (average)	4.35e-01	-9.09e-01	1.42e+00	0.86

Signif. codes: 0 '***' 0.001 '**' 0.01 '*' 0.05 '.' 0.1 ' ' 1
Sample Size Used: 22128
Simulations: 50000

Association between confounders and the LIFO brain network phenotype

Confounder	R^2	Pearson r	p-value	n in 40k
Age_sqr	0.2917	-0.5401	1.00E-300	39677
Age	0.2857	-0.5345	1.00E-300	39677
Age_qn	0.2810	-0.5301	1.00E-300	39677
Age_sqr_qn	0.2810	-0.5301	1.00E-300	39677
Age_sex_qn	0.1574	-0.3967	1.00E-300	39677
Age_sqr_sex_qn	0.1574	-0.3967	1.00E-300	39677
Age_sqr_sex	0.1305	-0.3613	1.00E-300	39677
Age_sex	0.1000	-0.3162	1.00E-300	39677
Sex	0.0673	-0.2594	1.00E-300	39677
Head_size	0.0276	0.1661	1.72E-243	39677
Head_size_qn	0.0274	0.1655	1.76E-241	39677
Table_pos_Y_qn	0.0087	-0.0934	1.37E-77	39677
Table_pos_Y_sqr_qn	0.0087	-0.0934	1.37E-77	39677
Table_pos_Y	0.0078	-0.0881	3.45E-69	39677
Table_pos_Y_sqr	0.0075	-0.0868	3.43E-67	39677
Site	0.0049	-0.0702	1.64E-44	39677
Structural_motion_qn	0.0042	-0.0649	2.45E-38	39677
Structural_motion	0.0041	-0.0640	3.01E-37	39677
Table_pos_Tab_sqr	0.0041	-0.0640	2.95E-37	39677
Table_pos_Tab	0.0041	0.0639	3.36E-37	39677
Table_pos_Tab_qn	0.0039	0.0622	2.84E-35	39677
Table_pos_Tab_sqr_qn	0.0039	-0.0622	2.84E-35	39677
Acquisition_date	0.0036	-0.0602	3.85E-33	39677
Table_pos_Z_sqr	0.0015	-0.0384	1.96E-14	39677
Structural_motion_sqr	0.0010	-0.0311	5.55E-10	39677
Table_pos_X_sqr_qn	0.0006	-0.0240	1.81E-06	39677
Table_pos_Z_sqr_qn	0.0003	-0.0186	0.000	39677
Table_pos_X_sqr	0.0002	-0.0133	0.008	39677
Table_pos_Z	0.0002	-0.0128	0.011	39677
Table_pos_X_qn	0.0002	-0.0124	0.014	39677
Table_pos_X	0.0001	-0.0117	0.019	39677
Table_pos_Z_qn	0.0001	0.0103	0.039	39677
