# Supplementary material for: The effects of genetic and modifiable risk factors on brain regions vulnerable to ageing and disease
Source: Nat Commun. 2024 Mar 27;15:2576. doi: 10.1038/s41467-024-46344-2 (PMC10973379; doi:10.1038/s41467-024-46344-2)
Supplement: Supplementary file 3 — Description of Additional Supplementary Files [file 41467_2024_46344_MOESM3_ESM.pdf]

Description of Additional Supplementary Files

File Name: Supplementary Data 1
Description: **Summary of significantly associated genetic clusters with the vulnerable 'last in, first out' brain network. Lead variant in bold.**
UK Biobank nIDP associations related to modifiable risk factors for dementia are also in bold; nIDP corresponding exactly to the best modifiable risk factors are underlined (based on https://genetics.opentargets.org/).
P-values are derived from a two-sided linear association test.

File Name: Supplementary Data 2
Description: **Significant associations between rs312238 (CD99) and non-imaging phenotypes (n=16,924) in the genotyped participants who had not been scanned (n=374,230)**

File Name: Supplementary Data 3
Description: **Extended Table 2. Details and associations with the vulnerable 'last in, first out' brain network for each modifiable risk factor (MRF) across 15 different categories.** 'Best' MRFs are in bold. P-values are derived from a two-sided linear association test.

File Name: Supplementary Data 4
Description: **Full list of variants in the MAPT region significantly associated with the vulnerable LIFO brain network (Cluster 5)**

File Name: Supplementary Data 5
Description: **Correlations between 'best' modifiable risk factors and the 6 confounders (bottom left of matrix: partial correlation; top right of matrix: full correlation).** Top Matrix: correlations; bottom matrix: corresponding P-values. Non-significant results are in grey.
