# Supplementary material for: The effects of genetic and modifiable risk factors on brain regions vulnerable to ageing and disease
Source: Nat Commun. 2024 Mar 27;15:2576. doi: 10.1038/s41467-024-46344-2 (PMC10973379; doi:10.1038/s41467-024-46344-2)
Supplement: Supplementary file 5 — Reporting Summary [file 41467_2024_46344_MOESM5_ESM.pdf]

nature portfolio

Corresponding author(s): Gwenaelle Douaud

Last updated by author(s): 02/02/2024

Reporting Summary

Nature Portfolio wishes to improve the reproducibility of the work that we publish. This form provides structure for consistency and transparency in reporting. For further information on Nature Portfolio policies, see our Editorial Policies and the Editorial Policy Checklist.

Statistics

For all statistical analyses, confirm that the following items are present in the figure legend, table legend, main text, or Methods section.

n/a	Confirmed	
☐	☒	The exact sample size (*n*) for each experimental group/condition, given as a discrete number and unit of measurement
☐	☒	A statement on whether measurements were taken from distinct samples or whether the same sample was measured repeatedly
☐	☒	The statistical test(s) used AND whether they are one- or two-sided<br>*Only common tests should be described solely by name; describe more complex techniques in the Methods section.*
☐	☒	A description of all covariates tested
☐	☒	A description of any assumptions or corrections, such as tests of normality and adjustment for multiple comparisons
☐	☒	A full description of the statistical parameters including central tendency (e.g. means) or other basic estimates (e.g. regression coefficient) AND variation (e.g. standard deviation) or associated estimates of uncertainty (e.g. confidence intervals)
☐	☒	For null hypothesis testing, the test statistic (e.g. *F*, *t*, *r*) with confidence intervals, effect sizes, degrees of freedom and *P* value noted<br>*Give P values as exact values whenever suitable.*
☒	☐	For Bayesian analysis, information on the choice of priors and Markov chain Monte Carlo settings
☒	☐	For hierarchical and complex designs, identification of the appropriate level for tests and full reporting of outcomes
☐	☒	Estimates of effect sizes (e.g. Cohen's *d*, Pearson's *r*), indicating how they were calculated

Our web collection on statistics for biologists contains articles on many of the points above.

Software and code

Policy information about availability of computer code

Data collection	Data were obtained from UK Biobank (available from UK Biobank upon data access application).
Data analysis	Data were analysed using the latest version of FSL at the time (FSL 6.0), and MATLAB (R2019-R2022). The mediation analysis was performed using version 4.5.0 of the R package mediation (https://cran.r-project.org/web/packages/mediation/mediation.pdf)

For manuscripts utilizing custom algorithms or software that are central to the research but not yet described in published literature, software must be made available to editors and reviewers. We strongly encourage code deposition in a community repository (e.g. GitHub). See the Nature Portfolio guidelines for submitting code & software for further information.

Data

Policy information about availability of data

All manuscripts must include a data availability statement. This statement should provide the following information, where applicable:

- Accession codes, unique identifiers, or web links for publicly available datasets
- A description of any restrictions on data availability
- For clinical datasets or third party data, please ensure that the statement adheres to our policy

All source data is available (upon data access application) from UK Biobank, and using VBM GM maps previously generated and made available in full: https://www.fmrib.ox.ac.uk/datasets/FLICA_ThickAreaVol/.
All the FLICA decomposition maps - including the LIFO grey matter network - in UK Biobank standard space, the UK Biobank grey matter template, scripts, and the

LIFO loadings for all of the participants are freely available on a dedicated webpage: open.win.ox.ac.uk/pages/douaud/ukb-lifo-flica/.

Research involving human participants, their data, or biological material

Policy information about studies with human participants or human data. See also policy information about sex, gender (identity/presentation), and sexual orientation and race, ethnicity and racism.

Reporting on sex and gender	We have made sure to use the term sex throughout, and have added this information in the Methods: "Sex was based on the population characteristics entry of UK Biobank. This is a mixture of the sex the NHS had recorded for the participant at recruitment, and updated self-reported sex. For the GWAS, both sex and genetic sex were used (the sample was excluded in case of a mismatch). "<br>Sex has been used as a confounder (sex, age × sex, age^2 × sex).
Reporting on race, ethnicity, or other socially relevant groupings	We examined SES via the Townsend Deprivation Index, but it had little bearing on the results, and thus was not considered as a confounding factor.<br>White/non-White ethnicity in UK Biobank is comparable with the UK general population.
Population characteristics	Age: 44-82 years, mean 64 ± 7 years<br>(M/F 47%-53%)
Recruitment	Participants recruited by UK Biobank: the participants in this prospective imaging study are healthier than the UK Biobank at large, which is itself healthier, less deprived, and better educated than the UK general population.
Ethics oversight	Human subjects: UK Biobank has approval from the North West Multi-centre Research Ethics Committee (MREC) to obtain and disseminate data and samples from the participants (http://www. ukbiobank.ac.uk/ethics/), and these ethical regulations cover the work in this study. Written informed consent was obtained from all participants. A statement on this is included in the paper.

Note that full information on the approval of the study protocol must also be provided in the manuscript.

Field-specific reporting

Please select the one below that is the best fit for your research. If you are not sure, read the appropriate sections before making your selection.

☒ Life sciences ☐ Behavioural & social sciences ☐ Ecological, evolutionary & environmental sciences

For a reference copy of the document with all sections, see nature.com/documents/nr-reporting-summary-flat.pdf

Life sciences study design

All studies must disclose on these points even when the disclosure is negative.

Sample size	Sample size was determined by data availability from UK Biobank. Exact samples sizes are listed in Methods and in Tables.
Data exclusions	For the genetic analyses, we applied filters to remove variants with minor allele frequency (MAF) below 0.1% and with an imputation information score below 0.3. We then kept only those participants estimated to have recent British ancestry using the sample quality control information provided centrally by UK Biobank (using the variable in.white.British.ancestry.subset in the file ukb_sqc_v2.txt); population structure can be a serious confound to genetic association studies, and this type of sample filtering is standard. The UK Biobank dataset also contains a number of close relatives (3rd cousin or closer). We therefore created a subset of nominally unrelated subjects. This follows similar procedures in our previous papers, e.g., Elliott et al. (2018).
Replication	For the genome-wide association study: we randomly split the samples into a discovery set with 2/3 of the samples (n = 22,128) and a replication set with 1/3 of the samples (n = 11,083). See Methods.
Randomization	N/A
Blinding	N/A

Reporting for specific materials, systems and methods

We require information from authors about some types of materials, experimental systems and methods used in many studies. Here, indicate whether each material, system or method listed is relevant to your study. If you are not sure if a list item applies to your research, read the appropriate section before selecting a response.

Materials & experimental systems

n/a	Involved in the study
☒	☐ Antibodies
☒	☐ Eukaryotic cell lines
☒	☐ Palaeontology and archaeology
☒	☐ Animals and other organisms
☐	☒ Clinical data
☒	☐ Dual use research of concern
☒	☐ Plants

Methods

n/a	Involved in the study
☒	☐ ChIP-seq
☒	☐ Flow cytometry
☐	☒ MRI-based neuroimaging

Clinical data

Policy information about clinical studies

All manuscripts should comply with the ICMJE guidelines for publication of clinical research and a completed CONSORT checklist must be included with all submissions.

Clinical trial registration	N/A
Study protocol	N/A
Data collection	N/A
Outcomes	N/A

Plants

Seed stocks	N/A: Bug in the new interactive PDF! Appears when *not* ticking the Plants box. Disappears when ticking it.
Novel plant genotypes	N/A
Authentication	N/A

Magnetic resonance imaging

Experimental design

Design type	N/A
Design specifications	N/A
Behavioral performance measures	N/A

Acquisition

Imaging type(s)	T1-weighted
Field strength	3T
Sequence & imaging parameters	se see Miller et al., Nature Neuroscience 2016 for a full list of the imaging parameters.
Area of acquisition	Whole brain

Diffusion MRI ☐ Used ☒ Not used

Preprocessing

Preprocessing software	FSL 6.0

Normalization	Non-linear registration was used based on the structural images (please see Miller et al., Nature Neuroscience 2016, and Alfaro-Almagro et al., Neuroimage 2018)
Normalization template	UK Biobank GM template based on 15K participants (provided).
Noise and artifact removal	Please see Alfaro-Almagro et al., Neuroimage 2018
Volume censoring	N/A

Statistical modeling & inference

Model type and settings	Regression/correlation (see Methods for full details).
Effect(s) tested	Effects of modifiable risk factors (with and without age/sex/head zise confounders), individually and in a unique model on the brain regions of interest.<br>Effects of genetic variants on the brain regions of interest.

Specify type of analysis: ☐ Whole brain ☐ ROI-based ☒ Both

Anatomical location(s): Based on the linear decomposition of grey matter structural variance using linked-ICA

Statistic type for inference<br>(See Eklund et al. 2016)	N/A
Correction	Bonferroni (genetics analyses: across genome, across non-imaging phenotypes; modifiable risk factors: across risk factors for individual analyses, across all combinations of every possible risk factor from each of the initial 15 risk factor categories for the combined study [unique model])

Models & analysis

n/a	Involved in the study
☒	☐ Functional and/or effective connectivity
☒	☐ Graph analysis
☒	☐ Multivariate modeling or predictive analysis
